# Intentional injuries in the Eastern Mediterranean Region, 1990–2015: findings from the Global Burden of Disease 2015 study

**DOI:** 10.1007/s00038-017-1005-2

**Published:** 2017-08-03

**Authors:** GBD 2015 Eastern Mediterranean Region Intentional Injuries Collaborators, Maziar Moradi-Lakeh, Raghid Charara, Charbel El Bcheraoui, Ibrahim Khalil, Ashkan Afshin, Nicholas J. Kassebaum, Michael Collison, Adrienne Chew, Kristopher J. Krohn, Farah Daoud, Danny Colombara, Nicholas Graetz, Michael Kutz, Haidong Wang, Foad Abd-Allah, Laith J. Abu-Raddad, Aliasghar Ahmad Kiadaliri, Muktar Beshir Ahmed, Khurshid Alam, Suliman Alghnam, Rajaa Al-Raddadi, Khalid A. Altirkawi, Nahla Anber, Palwasha Anwari, Leticia Avila-Burgos, Ashish Awasthi, Aleksandra Barac, Suzanne L. Barker-Collo, Neeraj Bedi, Zulfiqar A. Bhutta, Rohan Borschmann, Soufiane Boufous, Zahid A. Butt, Carlos A. Castañeda-Orjuela, Koustuv Dalal, Hadi Danawi, Diego De Leo, Samath D. Dharmaratne, Shirin Djalalinia, Kerrie E. Doyle, Alireza Esteghamati, André Faro, Maryam S. Farvid, Seyed-Mohammad Fereshtehnejad, Florian Fischer, Tsegaye Tewelde Gebrehiwot, Reyna A. Gutiérrez, Nima Hafezi-Nejad, Randah Ribhi Hamadeh, Samer Hamidi, Josep Maria Haro, Delia Hendrie, Guoqing Hu, Jost B. Jonas, Peter Njenga Keiyoro, Yousef Saleh Khader, Ejaz Ahmad Khan, Jagdish Khubchandani, Jacek A. Kopec, Heidi J. Larson, Asma Abdul Latif, Cheru Tesema Leshargie, Raimundas Lunevicius, Mohammed Magdy Abd El Razek, Azeem Majeed, Reza Malekzadeh, Ziad A. Memish, Tuomo J. Meretoja, Ted R. Miller, Shafiu Mohammed, Carla Makhlouf Obermeyer, Felix Akpojene Ogbo, Michael Robert Phillips, Farshad Pourmalek, Mostafa Qorbani, Amir Radfar, Anwar Rafay, Afarin Rahimi-Movaghar, Vafa Rahimi-Movaghar, Rajesh Kumar Rai, David Laith Rawaf, Salman Rawaf, Satar Rezaei, Mohammad Sadegh Rezai, Gholamreza Roshandel, Mahdi Safdarian, Saeid Safiri, Payman Salamati, Abdallah M. Samy, Benn Sartorius, Soraya Seedat, Sadaf G. Sepanlou, Masood Ali Shaikh, Badr H. A. Sobaih, Karen M. Tabb, Arash Tehrani-Banihashemi, Mohamad-Hani Temsah, Abdullah Sulieman Terkawi, Roman Topor-Madry, Kingsley Nnanna Ukwaja, Olalekan A. Uthman, Mehdi Yaseri, Naohiro Yonemoto, Mustafa Z. Younis, Aisha O. Jumaan, Theo Vos, Simon I. Hay, Mohsen Naghavi, Christopher J. L. Murray, Ali H. Mokdad

**Affiliations:** Institute for Health Metrics and Evaluation, 2301 5th Avenue, Suite 600, Seattle, WA 98121 USA

**Keywords:** Intentional injuries, Eastern mediterranean region, Burden of disease

## Abstract

**Objectives:**

We used GBD 2015 findings to measure the burden of intentional injuries in the Eastern Mediterranean Region (EMR) between 1990 and 2015.

**Methods:**

The Global Burden of Disease (GBD) study defines intentional injuries as a combination of self-harm (including suicide), interpersonal violence, collective violence (war), and legal intervention. We estimated number of deaths, years of life lost (YLLs), years lived with disability (YLDs), and disability-adjusted life years (DALYs) for each type of intentional injuries.

**Results:**

In 2015, 28,695 individuals (95% UI: 25,474–37,832) died from self-harm, 35,626 (95% UI: 20,947–41,857) from interpersonal violence, and 143,858 (95% UI: 63,554–223,092) from collective violence and legal interventions. In 2015, collective violence and legal intervention was the fifth-leading cause of DALYs in the EMR and the leading cause in Syria, Yemen, Iraq, Afghanistan, and Libya; they account for 49.7% of total DALYs in Syria.

**Conclusions:**

Our findings call for increased efforts to stabilize the region and assist in rebuilding the health systems, as well as increasing transparency and employing preventive strategies to reduce self-harm and interpersonal injuries.

**Electronic supplementary material:**

The online version of this article (doi:10.1007/s00038-017-1005-2) contains supplementary material, which is available to authorized users.

## Introduction

Intentional injuries accounted for more than 1.4 million deaths and about 4% of total years of life lost (YLLs) in 2015 globally (GBD 2015 Mortality and Causes of Death Collaborators 2016). The Global Burden of Disease (GBD) study defines intentional injuries as a combination of self-harm (including suicide), interpersonal violence (such as homicide and physical and sexual assault), collective violence (or war), and legal intervention (such as police enforcement). Intentional injuries are important because, in theory, intentional injuries can be avoided by intention of human beings; this is not the case for most of the other injuries and diseases. In spite of this fact, about 30% of all global deaths from injuries in 2015 were intentional, and suicide and homicide were among the top 10 leading causes of deaths in 15–49-year-old individuals (GBD 2015 Mortality and Causes of Death Collaborators 2016; Institute for Health Metrics and Evaluation (IHME) 2017). Conflict obviously increases deaths and injuries on the battlefield, and also affects health due to the displacement of populations, the breakdown of health and social services, and the heightened risk of disease transmission (Murray et al. 2002).

The Eastern Mediterranean Region (EMR) has had several conflicts and unrests in the past years; such events have huge impact on all types of intentional injuries. A study in Tunisia showed an increase by 1.7 times in self-harm and 1.3 times in homicide after the Tunisian Revolution in 2011 (Ben Khelil et al. 2016). The effect of conflicts and social unrest on collective violence and legal intervention is obvious. Intentional injuries such as suicide are usually underreported due to cultural and religious norms. Previous studies reported on the burden of disease in the region but did not focus on intentional injuries (Mokdad et al. 2014, 2016). To better estimate the burden of intentional injures, we used the GBD 2015 study to report the mortality, morbidity, and burden of intentional injuries in EMR countries from 1990 to 2015.

## Methods

The 2015 Global Burden of Disease (GBD 2015) covered 249 causes of death and 310 non-fatal diseases and injuries. GBD 2015 reported the burden for 195 countries or territories, 21 regions, and seven super-regions for the 1990–2015 time period. The general methodology of GBD 2015 has been detailed elsewhere (GBD 2015 DALYs and HALE Collaborators 2016; GBD 2015 Disease and Injury Incidence and Prevalence Collaborators 2016; Haagsma et al. 2016; GBD 2015 Mortality and Causes of Death Collaborators 2016).

The EMR contains 22 countries: Afghanistan, Bahrain, Djibouti, Egypt, Iran, Iraq, Jordan, Kuwait, Lebanon, Libya, Morocco, Oman, Pakistan, Palestine, Qatar, Saudi Arabia, Somalia, Sudan, Syria, Tunisia, United Arab Emirates, and Yemen.

We classified intentional injuries as self-harm, interpersonal violence (which includes physical violence by firearm, sharp objects, or other means) and “collective violence and legal intervention.” Collective violence includes wars, terrorism, and other violent political conflicts within or between states, state-perpetrated violence (such as genocide, repression, disappearances, torture, and other abuses of human rights), and organized violent crimes such as gang warfare (WHO 2014). We estimated fatal and non-fatal intentional injuries to calculate disability-adjusted life years (DALYs).

### Mortality input data and cause of death models

We estimated injury mortality from different sources (vital registration, verbal autopsy, mortality surveillance, censuses, surveys, and police record data). More information on input sources of data are available elsewhere (GBD 2015 Mortality and Causes of Death Collaborators 2016). We used the standard CODEm modeling approach to estimate deaths due to intentional causes of injuries, excluding collective violence and legal intervention. This cause was modeled solely outside of the CODEm process as fatal discontinuities estimation (or mortality shock regression).

The output mortality estimates were used to calculate years of life lost (YLLs) for each cause of death.

### Cause-of-injury incidence; input data and modeling

The majority of incidence data exist at the external cause‐of‐injury level, i.e., E-codes. Incidence for cause‐of‐injury categories was modeled using DisMod‐MR 2.1 for self-harm and interpersonal violence.

DisMod‐MR 2.1 is a descriptive epidemiological meta‐regression tool that uses the integrative systems modeling approach to produce simultaneous estimates of disease incidence, prevalence, remission, and mortality. Multiple datasets from hospital, emergency/outpatient departments, and survey datasets are fed into these incidence models. We separately estimated inpatient and outpatient injuries.

To estimate incidence from the shock cause‐of‐injury categories (collective violence and legal intervention), the mortality rate for these cause‐of‐injury categories was multiplied by the average country‐year‐age‐sex‐specific incidence‐to‐mortality ratios within several cause‐of-injury categories that likely exhibit similar case fatality ratios (such as road injuries, fires, interpersonal violence, and other unintentional injuries) (GBD 2015 Disease and Injury Incidence and Prevalence Collaborators 2016; Haagsma et al. 2016).

We imposed a hierarchy to select the nature-of-injury category that leads to the largest burden when an individual experiences multiple injuries. Then, we separated matrices (for inpatient and outpatient injuries) to estimate the proportions of incident cases in each of the cause-of-injury categories that resulted in each of the nature-of-injury categories (N-codes). We produced incidence of inpatient and outpatient injuries by cause and nature of injury. Then we estimated short-term disability by nature-of-injury category for all incident cases of inpatient and outpatient injuries. We estimated the average duration for each nature-of-injury category and derived short-term prevalence by multiplying incidence and duration.

We then applied DisMod-MR 2.1 to estimate the long-term prevalence for each combination of cause-of-injury and nature-of-injury from incidence and the long-term mortality risk in cases with long-term disability. After correction for comorbidity with other non-fatal diseases, YLDs were calculated as prevalence times a disability weight. More details on the process are available elsewhere (GBD 2015 Disease and Injury Incidence and Prevalence Collaborators 2016; Haagsma et al. 2016).

Disability-adjusted life years (DALYs) were calculated for each type of injury through summation of YLLs and YLDs (GBD 2015 DALYs and HALE Collaborators 2016).

### Uncertainty

We have propagated uncertainty from all different sources such as input data or adjustment process, using standard GBD methods of repeating all calculations 1000 times, each time drawing from distributions rather than point estimates for all the relevant parameters in our models. We then used 2.5th and 97.5th percentiles as the lower and upper bounds of the 95% uncertainty interval (UI). For the injury mortality estimates, the estimation of model uncertainty is inherent to the ensemble modeling method. Some of the rates that we present are age-standardized using the GBD standard population (GBD 2015 DALYs and HALE Collaborators 2016; GBD 2015 Disease and Injury Incidence and Prevalence Collaborators 2016; Haagsma et al. 2016; GBD 2015 Mortality and Causes of Death Collaborators 2016).

## Results

In 2015 in the EMR, 28,695 individuals (95% UI: 25,474–37,832) died from self-harm, 35,626 individuals (95% UI: 20,947–41,857) from interpersonal violence, and 143,858 individuals (95% UI: 63,554–223,092) from collective violence and legal intervention. These numbers show a significant increase from those in 1990, accounting for a 100% increase in self-harm, 152% increase for interpersonal violence, and 1027% increase for collective violence and legal intervention. In comparison, during the same time, the number of deaths in other parts of the world due to self-harm and interpersonal violence increased by 19 and 12%, respectively, and decreased by 67% for collective violence and legal intervention. Male to female ratio of deaths in 2015 in EMR was 2.4 for self-harm, 4.0 for interpersonal violence, and 3.3 for collective violence and legal interventions.

Among the total number of deaths in the EMR due to interpersonal violence, firearms and sharp objects accounted for 14,158 (8782–17,306) and 7195 (3758–10,864) deaths, respectively, in 2015.

**Fig. 1:**
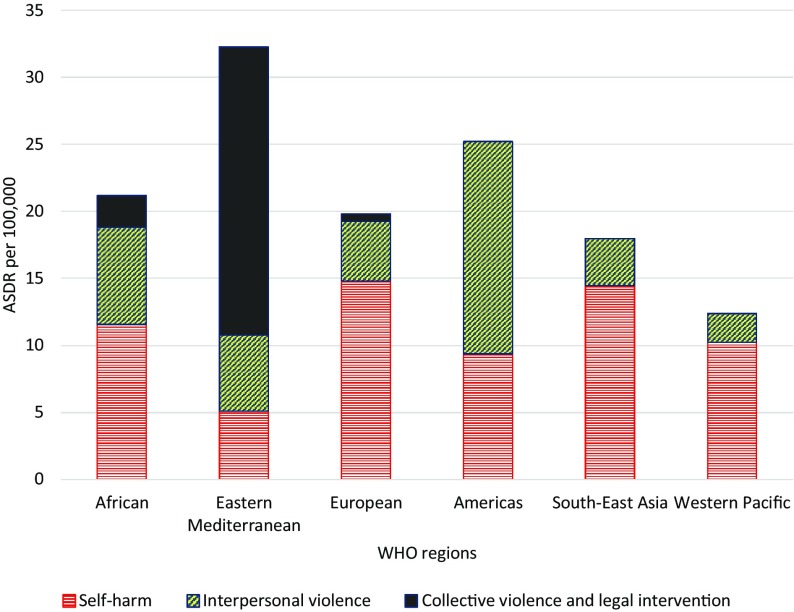
Age-standardized death rates (ASDR) per 100,000 from self-harm, interpersonal violence, and collective violence and legal intervention (Global Burden of Disease 2015 study, WHO Regions, 2015)

In 2015, the age-standardized death rate (ASDR) of self-harm in the EMR (5.1 per 100,000, 95% UI: 4.6–6.6) was lower than that of all other World Health Organization regions (Fig. 1), and the ASDR of interpersonal violence in the EMR (5.7 per 100,000, 95% UI: 3.8–6.6) was less than the Americas and African regions, and higher than the other WHO regions. However, the ASDR of collective violence and legal intervention (21.5 per 100,000 population, 95% UI: 9.3–33.5) was much higher in the EMR than other WHO regions (Fig. 1). The patterns for all-age death rates were relatively similar.

**Fig. 2:**
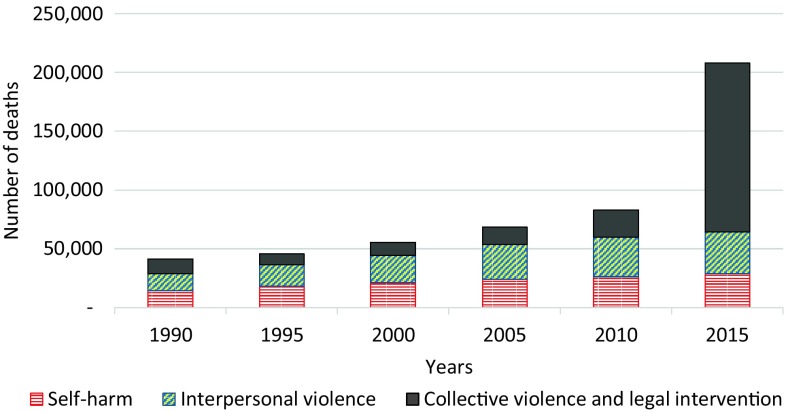
Number of deaths due to intentional injuries (Global Burden of Disease 2015 study, Eastern Mediterranean Region, 1990–2015)

Total number of deaths due to intentional injuries (self-harm, interpersonal violence, collective violence and legal intervention) showed an increasing trend between 1990 and 2015 in the EMR (Fig. 2); the most important increase was observed between 2010 and 2015, and was mainly due to collective violence and legal intervention.

**Fig. 3:**
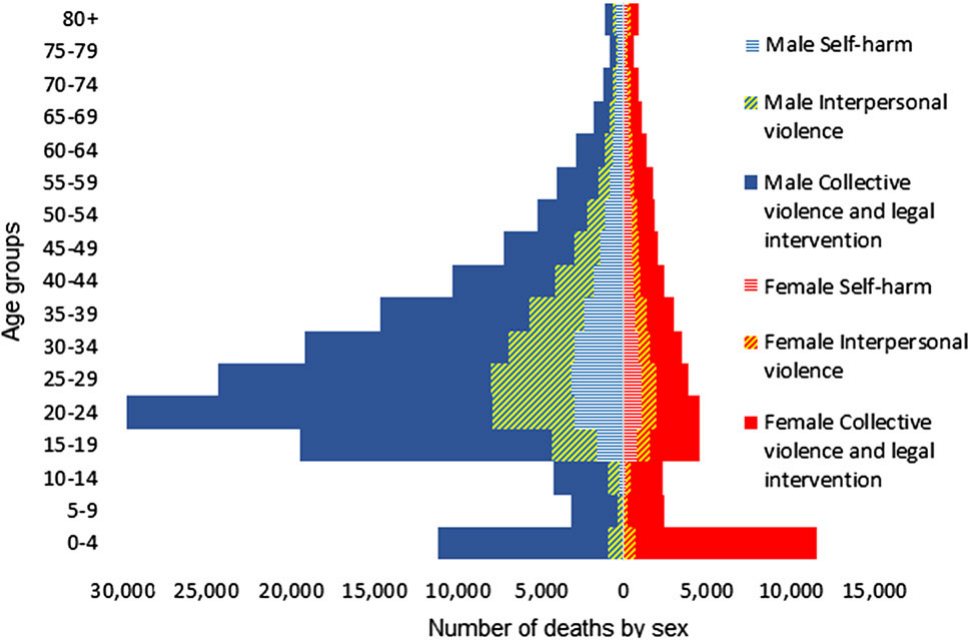
Age–sex distribution of deaths due to intentional injuries (Global Burden of Disease 2015 study, Eastern Mediterranean Region, 2015)

Figure 3 shows the age–sex distribution of deaths due to different types of intentional injuries in the EMR (2015). Males had higher rates, except for the youngest and oldest age groups. Among males, the mortality was considerably higher in people aged 20–24 years. Girls under 5 years old had a higher mortality from intentional injuries (Fig. 3)

**Fig. 4:**
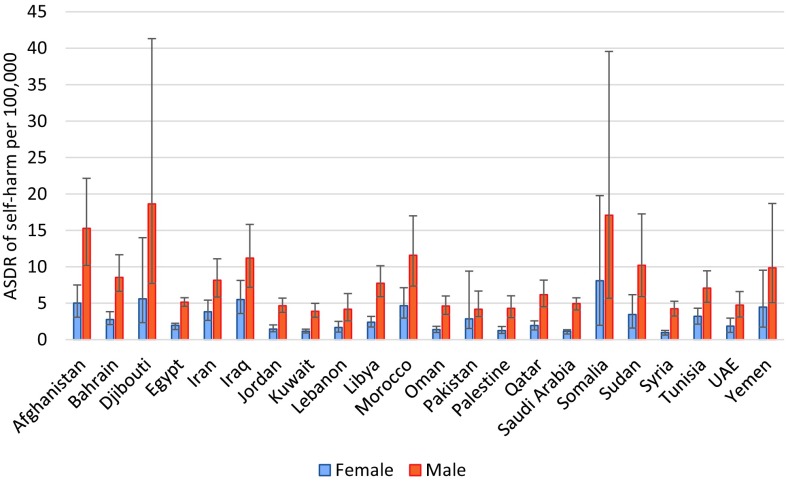
Age-standardized death rates (ASDR) from self-harm per 100,000 men and women in the countries of the Eastern Mediterranean Region (Global Burden of Disease 2015 study, Eastern Mediterranean countries, 2015)

**Fig. 5:**
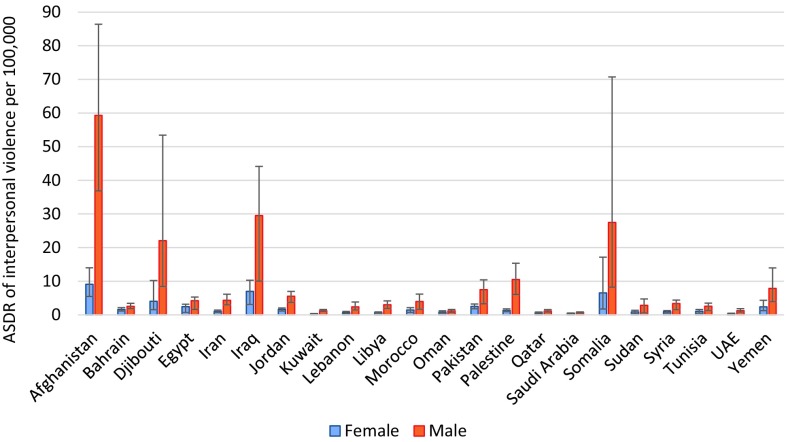
Age-standardized death rates (ASDR) from interpersonal violence (per 100,000 population), for men and women in the countries of the Eastern Mediterranean Region (Global Burden of Disease 2015 study, Eastern Mediterranean countries, 2015)

**Fig. 6:**
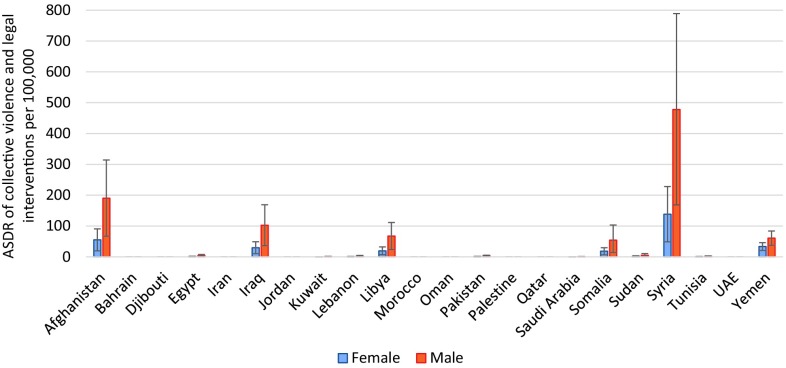
Age-standardized death rates (ASDR) for collective violence and legal intervention (per 100,000 population), for men and women in the countries of the Eastern Mediterranean Region (Global Burden of Disease 2015 study, Eastern Mediterranean countries, 2015)

The highest ASDR of self-harm was observed in men of Djibouti, Somalia, and Afghanistan, and women of Somalia, Djibouti, and Iraq (Fig. 4). Afghanistan, Iraq, and Somalia had the highest ASDR of interpersonal violence for both men and women (Fig. 5). The highest ASDR of collective violence and legal interventions was observed in Syria, Afghanistan, and Iraq for both sexes (Fig. 6). Syria had an ASDR of 138.2 per 100,000 for women (95% UI: 48.8–228.2) and 478.0 per 100,000 for men (168.8–789.0) for collective violence and legal intervention in 2015.

DALYs from self-harm, interpersonal violence, and collective violence and legal intervention in 2015 totaled 1,425,494 (95% UI: 1,258,222–1901,949), 1,997,224 (95% UI: 1,184,027–2,325,345), and 10,107,643 (95% UI: 5,381,404–14,787,629), respectively. YLLs were the main component of DALYs for all kinds of intentional injuries; YLLs accounted for more than 98.5% of DALYs for self-harm and interpersonal violence and 85.9% of DALYs for collective violence and legal intervention.

Lebanon had the highest median percentage of total DALYs from intentional injuries, followed by Iraq, Afghanistan, and Palestine. The lowest median percentage was in Saudi Arabia, followed by Egypt and Oman (e-Table 1).

In 2015, collective violence and legal intervention was the fifth-leading cause of DALYs in the EMR, the first-leading cause of DALYs in five countries (Syria, Yemen, Iraq, Afghanistan, and Libya), and the second-leading cause in Lebanon. In Syria, 49.7% of total DALYs (95% UI: 30.8–62.2) in 2015 were due to collective violence and legal intervention.

## Discussion

Our study showed that the burden of intentional injuries is increasing rapidly in the EMR. It is not a surprise that the burden of collective violence and legal intervention has increased dramatically in the last few years and is currently higher than rest of the world due to the unrest in the region. However, our study showed a rise in self-harm and interpersonal violence during the same time period that was much faster than in other parts of the world. Clearly the unrest and conflicts are causing deaths due to collective violence and legal interventions, but they are also correlated with increased burden from self-harm and other diseases (Murray et al. 2002; Ben Khelil et al. 2016). All countries with the highest mortality rates of self-harm and interpersonal violence (Afghanistan, Somalia, Djibouti, and Iraq) have been affected by multiple episodes of civil or inter-state wars and social unrest, as well as terrorism during 1990–2015. Our study calls for efforts to stabilize the region politically and reduce the burden of disease due to the current situations.

Young men are the most typical victims of intentional injuries, especially interpersonal and collective violence. This pattern is relatively similar to other regions of the world (Degutis 2013). Girls and boys under 5 years old had a large share of total deaths due to collective violence. This might be due to their generally higher vulnerability in emergency situations. Like the pre-conflict state, mortality is higher among children than individuals over 5 years during a conflict; however, individuals over 5 are usually affected more than young children. In other words, while the general mortality rate of children under 5 is around ten times that of individuals over 5 in pre-conflict states, it decreases to around double during a conflict state (Guha-Sapir and Panhuis 2004). The age pattern of deaths due to collective violence might be related to several factors such as type of war (for instance, civil wars versus inter-state wars) and main types of arms involved (individual light weapons compared to heavy artillery and weapons of mass destruction).

The absolute and relative importance of direct injuries from collective violence and legal intervention has increased significantly in the region in recent years. Although the region has experienced several conflicts in recent decades, the Syrian war has increased deaths and burden of collective violence significantly in recent years. The total burden imposed by war is certainly higher because it also indirectly increases death and disability from other diseases. On the other hand, the number of people who died from a war is not limited to the time period of its occurrence. Previous studies show that several years after termination of wars, people are at higher risk of death due to its consequences such as remaining land mines. In addition, some people suffer from the long-term complications of injuries such as amputations and spinal cord injury for years after war and are at risk of premature death for the same reasons (Mousavi et al. 2014).

In this study, mortality and burden of self-harm in the EMR were lower than in other regions of the world. Although religious and cultural beliefs might have contributed to these low rates, the burden of self-harm also might be affected by cultural and religious barriers, social stigma, and legal punishments that encourage victims, families, and governments to hide the information (Malakouti et al. 2015a). Methods of suicide in the EMR show some differences from other regions of the world. Hanging and poisoning are the most common methods of suicide; however, there are also differences between and even within countries (Morovatdar et al. 2013). These are important because there are specific interventions to prevent each type of suicide. Many of the preventive strategies focus on finding individuals who are at higher risk of suicide attempts, such as those with mental illness after discharge from a hospital (Ghanbari et al. 2015). In Iran, some trials have been done to integrate suicide prevention services into primary health care (PHC), which increases universal access to and sustainability of these services (Malakouti et al. 2015b, c).

Interpersonal violence is an important cause of DALYs in some of the countries of the EMR, especially Afghanistan, Iraq, Somalia, and Djibouti. It is not always easy to separate interpersonal violence from collective violence, especially when a civil war is taking place. In this study, firearms had a major contribution to total deaths from interpersonal injuries. Although having a gun is illegal in most of the countries of the region, having access to weapons is not difficult in countries such as Afghanistan and Iraq (after being involved in civil wars for several years).

Our study has some limitations. First, reports on intentional injuries (especially self-harm and legal intervention) are subject to underreporting or even being covered up in many countries. We used the general GBD methodology to address underreporting of deaths; however, underreporting might be different for specific causes of deaths. Second, the number of war victims is not usually accurate due to poor health information systems and political considerations of reporting; many of the countries involved in conflicts do not have a reliable health information system even in their pre-conflict states. Third, we did not evaluate the indirect effects of collective violence (war) on health workforce, infrastructure, and food security. These factors can considerably increase the attributable burden to war. Finally, we did not account for the impact of the influx of refugees on the health systems and disease burden of the host countries.

### Conclusions

Our study documented the burden of intentional injuries due to the conflicts and unrest in the EMR. Moreover, we showed an increased burden from other intentional injuries at the same time. Our findings call for increased efforts to stabilize the region and assist in rebuilding the health systems, as well as increasing transparency and employing preventive strategies to reduce self-harm and interpersonal injuries.

## Electronic supplementary material

Below is the link to the electronic supplementary material.

Supplementary material 1 (DOCX 18 kb)

Supplementary material 2 (XLSX 21 kb)
