# Supplementary material for: Intentional injuries in the Eastern Mediterranean Region, 1990–2015: findings from the Global Burden of Disease 2015 study
Source: Int J Public Health. 2017 Aug 3;63(Suppl 1):39–46. doi: 10.1007/s00038-017-1005-2 (PMC5973968; doi:10.1007/s00038-017-1005-2)
Supplement: Supplementary file 1 — Supplementary material 1 (DOCX 18 kb) [file 38_2017_1005_MOESM1_ESM.docx]

International Journal of Public Health

Electronic Supplementary Material

**Article title:**

Intentional injuries in the Eastern Mediterranean Region, 1990–2015: Findings from the Global Burden of Disease 2015 study

**Authors:**

GBD 2015 Eastern Mediterranean Region Intentional Injuries Collaborators

**Corresponding author:**

Ali H. Mokdad

Institute for Health Metrics and Evaluation, University of Washington, Seattle, WA, United States

Email: [mokdaa@uw.edu](mailto:mokdaa@uw.edu)

e-Table 1. Percent share of intentional injuries from total disability-adjusted life years (DALYs)

in the Eastern Mediterranean Region countries (Findings from the Global Burden of Disease 2015 study, Eastern Mediterranean countries, 1990–2015)

| Country | Mean | Median | Minimum | Maximum |
| --- | --- | --- | --- | --- |
| Afghanistan | 6.3% | 5.0% | 3.7% | 14.3% |
| Bahrain | 2.9% | 2.8% | 2.3% | 3.7% |
| Djibouti | 1.5% | 1.5% | 0.8% | 2.1% |
| Egypt | 0.8% | 0.9% | 0.3% | 1.4% |
| Iran | 2.6% | 2.6% | 1.6% | 3.2% |
| Iraq | 7.8% | 6.0% | 4.2% | 16.1% |
| Jordan | 2.1% | 1.8% | 1.5% | 3.7% |
| Kuwait | 4.3% | 2.7% | 2.1% | 13.1% |
| Lebanon | 12.6% | 11.6% | 8.4% | 22.4% |
| Libya | 3.7% | 1.9% | 1.4% | 12.5% |
| Morocco | 1.4% | 1.5% | 1.0% | 1.8% |
| Oman | 0.9% | 0.9% | 0.5% | 1.3% |
| Pakistan | 1.0% | 0.9% | 0.6% | 1.6% |
| Palestine | 5.5% | 4.9% | 3.7% | 8.9% |
| Qatar | 2.1% | 2.0% | 1.8% | 2.4% |
| Saudi Arabia | 0.8% | 0.8% | 0.5% | 1.1% |
| Somalia | 2.2% | 2.1% | 1.1% | 3.8% |
| Sudan | 1.7% | 1.7% | 1.1% | 2.3% |
| Syria | 9.4% | 1.2% | 1.0% | 50.2% |
| Tunisia | 1.2% | 1.2% | 0.8% | 1.5% |
| UAE | 1.9% | 2.0% | 1.6% | 2.1% |
| Yemen | 3.5% | 1.4% | 0.7% | 14.6% |
